# Diagnostic aid to subepidermal calcified nodule with dermoscopy and reflectance confocal microscopy: a case report

**DOI:** 10.1186/s12887-023-03913-6

**Published:** 2023-03-03

**Authors:** Xiaomei Zhu, Xiaoyan Zhang, Kun Yang

**Affiliations:** Department of Dermatology and Venereology, Tongji Hospital, Tongji Medical College, Huazhong University of Science and Technology, 1095 Jiefang Avenue, Wuhan, 430030 China

**Keywords:** Case report, Subepidermal calcified nodule, Differential diagnosis, Dermoscopy, Reflectance confocal microscopy

## Abstract

**Background:**

Subepidermal calcified nodule (SCN) is a type of calcinosis cutis that usually occurs in children. The lesions in the SCN resemble those of other skin diseases, such as pilomatrixoma, molluscum contagiosum, and juvenile xanthogranuloma, leading to a high rate of misdiagnoses. Noninvasive in vivo imaging techniques, represented by dermoscopy and reflectance confocal microscopy (RCM), have dramatically accelerated skin cancer research over the past decade, and their applications have greatly expanded into other skin disorders. However, the features of an SCN in dermoscopy and RCM have yet to be reported previously. Combining these novel approaches with conventional histopathological examinations is a promising method for increasing diagnostic accuracy.

**Case presentation:**

We report on a case of SCN of the eyelid diagnosed with the aid of dermoscopy and RCM. A 14-year-old male patient who presented with a painless yellowish-white papule on his left upper eyelid was previously diagnosed with a common wart. Unfortunately, treatment with recombinant human interferon gel was not effective. To achieve a correct diagnosis, dermoscopy and RCM were performed. The former showed closely grouped multiple yellowish-white clods surrounded by linear vessels, and the latter exhibited hyperrefractile material nests at the dermal–epidermal junction level. The alternative diagnoses were, therefore, excluded because of in vivo characterizations. Subsequent surgical excision, histological examination, and von Kossa staining were performed. Pathology showed hyperkeratosis of the epidermis, a downward-directed basal-layer expansion, and small amorphous basophilic deposits scattered throughout the papillary dermis. The von Kossa staining confirmed calcium deposits in the lesion. An SCN was then diagnosed. During the 6-month follow-up, no relapse was observed.

**Conclusions:**

Patients with SCN could benefit from dermoscopy and RCM, which help achieve an accurate diagnosis. Clinicians should consider the possibility of an SCN for an adolescent patient with painless yellowish-white papules.

## Background

Subepidermal calcified nodule (SCN) is a subtype of calcinosis cutis, presenting as a solitary, hard, yellowish-white nodule [1]. Patients with SCN can phenotypically mimic and are often misdiagnosed with pilomatrixoma, molluscum contagiosum, and juvenile xanthogranuloma. Histological confirmation was the only practical option for the diagnosis of SCN when the lesions were equivocal on conventional naked-eye examination. The applications of noninvasive imaging techniques such as dermoscopy and reflectance confocal microscopy (RCM) have expanded following advances in the field of skin cancer [2]. These novel techniques can decrease unnecessary surgical biopsies and broaden treatment options. Here, we report a typical case of eyelid SCN and describe its dermoscopy and RCM findings to provide insight into novel approaches to differential diagnosis and management in the future.

## Case presentation

**Fig. 1:**
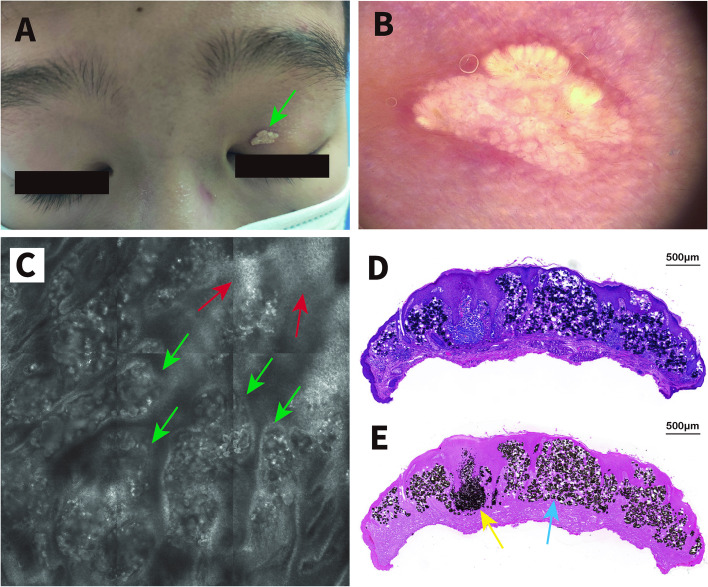
**A** Clinical presentation of the lesion. A yellowish-white papule on the patient's left upper eyelid; **B** Dermatoscopy of the lesion shows closely grouped multiple yellowish-white clods surrounded by linear vessels (× 20 magnification); **C** RCM imaging of the lesion shows nests of hyperrefractile material at the level of the dermal–epidermal junction (green arrows) and irregularly hyperplastic spinous layer (red arrows) (VivaScope 1500, mosaic, 0.5 × 0.5 mm); **D** Histopathology of the lesion shows small amorphous basophilic deposits scattered throughout the dermis with small numbers of lymphocytes, hyperkeratosis of the epidermis, a thickened stratum spinosum, and a downward-directed basal-layer expansion (HE stain, scale bar = 500 μm); **E** Von Kossa stain demonstrating numerous black calcium deposits either in the form of small amorphous calcium (blue arrows) or in the form of fine granules (yellow arrows). (von Kossa stain, scale bar = 500 μm)

A 14-year-old previously healthy male patient presented to our hospital with a painless yellowish-white papule on the left upper eyelid (Fig. 1A). The papule appeared five years previously and gradually became larger without ulceration or bleeding. Before being referred to our hospital, the patient was diagnosed with a common wart. However, treatment with recombinant human interferon gel was not effective. No history of trauma, local skin lesions, or familial similar skin lesions could be found. Cutaneous examination revealed a solitary yellowish-white papule approximately 5 mm in diameter on the upper left eyelid. Dermoscopy showed closely grouped multiple yellowish-white clods surrounded by linear vessels (Fig. 1B). RCM imaging showed nests of hyperrefractile material at the dermal–epidermal junction level and irregular hyperplasia of the epidermis (Fig. 1C). Surgical excision, the most common treatment option for SCN, was performed. A further histopathological examination revealed small amorphous basophilic deposits scattered throughout the papillary dermis with small numbers of lymphocytes, hyperkeratosis of the epidermis, a thickened stratum spinosum, and a downward-directed basal-layer expansion. Neither ghost cells, a feature of pilomatrixoma, nor a granular layer of keratohyalin granules, characteristic of epidermal cysts, were found (Fig. 1D). The presence of calcium deposits was confirmed by von Kossa staining (Fig. 1E). The levels of calcium, phosphorus and parathyroid hormones in the blood were normal. A diagnosis of eyelid SCN was made based on the above examination. The patient remained recurrence-free for the entire follow-up period (6 months).

## Discussion and conclusions

Calcinosis cutis can be classified into five major types: dystrophic calcification, metastatic calcification, iatrogenic calcification and traumatic calcinosis cutis, calciphylaxis, and idiopathic calcification [3]. SCN is a rare subtype of idiopathic calcification that usually occurs in healthy individuals and is unrelated to tissue damage, systemic disease, or side effects of treatments. To date, only over a hundred cases of SCN have been reported. The incidence of SCN among men is approximately twice that among women. Seventy-two percent of patients are younger than 18 years old. The lesions present asymptomatic, painless, hard, and freely mobile nodules and can be single (82%) or multiple (18%) [4]. In a few cases, the papule may hemorrhage [5]. Due to the rarity of eyelid SCN, physicians are unfamiliar with this diagnosis, and patients often experience misdiagnosis.

Beyond this, however, its morphological similarities with other skin diseases make SCN challenging to diagnose. It is frequently misdiagnosed as pilomatrixoma, juvenile xanthogranuloma, and molluscum contagiosum. While the naked-eye examination is unreliable in some cases, noninvasive in vivo imaging improves diagnostic accuracy. Dermoscopy is one of the primary imaging modalities used to diagnose cancerous skin lesions, such as melanoma or basal cell carcinoma. Over the last several years, dermoscopy has increasingly been used in the context of general dermatological disorders [6]. RCM is another novel technology that could also provide noninvasive, in vivo imaging of the skin at a near-histological resolution. Here, we showed, for the first time, images of eyelid SCN under dermoscopy and RCM. Dermoscopy showed closely grouped multiple yellowish-white clods surrounded by linear vessels, and RCM showed nests of hyperrefractile material at the dermal–epidermal junction level. These findings may be helpful in the differential diagnosis of SCN and other similar entities (Table 1). Moreover, hematoxylin–eosin (HE) staining showed basophilic materials in a finely granular or lumpy form, and von Kossa staining showed black calcium deposits in the dermis. It has been reported that the histopathological patterns of SCN in children and elderly patients are different [7]. Young patients often show multiple, small calcified bodies within the dermis surrounded by foreign-body giant cells and lymphoplasmacytic chronic inflammation. In contrast, elderly patients present lesions characterized by a single, large, well-demarcated amorphous calcified deposit surrounded by fibrous tissue without chronic inflammation or foreign body reaction. In agreement with this, we found multiple calcium nests in the dermis of this patient. However, it is still being determined why there are two forms of calcified deposits. Further studies are needed.

**Table 1:** Dermoscopic, reflectance confocal microscopic (RCM) and histopathological features of SCN and its differential diagnoses

Skin Disorders	Dermoscopy	RCM	Histopathology
SCN	Closely grouped multiple yellowish-white clods surrounded by linear vessels*	Hyperrefractile material nets at the dermal-epidermal junction level and irregular hyperplasia of the epidermis*	Small amorphous basophilic deposits scattered throughout the papillary dermis with small numbers of lymphocytes, hyperkeratosis of the epidermis, a thickened stratum spinosum, and a downward-directed basal-layer expansion*
Molluscum Contagiosum	A polylobular, white-yellow, amorphous structure in the center with a surrounding crown of vessels that do not cross the centers of the lobules [8]	A round, well-circumscribed lesion with central round cystic areas filled with brightly refractile material [9]	The characteristic molluscum bodies [9]
Juvenile Xanthogranuloma	Setting sun appearance, clouds of paler yellow globules, linear and branched vessels, and whitish streaks [10]	Epidermal normal honeycomb pattern, dome-shaped lesion, dilated dermal papillae at dermal-epidermal junction filled with clusters of roundish, large, multinucleated, and hyperrefractile atypical cells corresponding to Touton cells [11]	Vacuolated cells, xanthomatized cells, spindle-shaped cells, and oncocytic cells The histiocytic infiltrations in the papillary dermis and the reticular dermis [10]
Common Warts	Multiple densely packed papillae with a central red dot or loop, surrounded by a whitish halo; hemorrhages [12]	Elongated and enlarged dermal papillae containing dilated capillary vessels [6]	Elongated and enlarged dermal papillae containing dilated capillary vessels; koilocytes [6]
Pilomatrixoma	White and/or yellow homogeneous areas shaped and distributed irregularly (corresponding histopathologically to calcification or keratin masses), white streaks, reddish homogeneous areas, hairpin vessels, and linear irregular vessels [13]	Not reported	Basaloid cells, calcification and ghost (phantom, shadow) cells [14]
BCC (MAY)	multiple aggregated yellow-white globules [15]	A well-defined tumor with hyperreflective amorphous areas [15]	Tumor islands with palisading and clefting and calcium deposits [15]
MICC	round white homogeneous lesions, central crusts [16]	Not reported	Basophilic materials in superficial dermis [16]

*The findings of this study

BCC (MAY), Basal cell carcinoma with multiple aggregated yellow-white (MAY) globules, *MICC* Milia-like idiopathic calcinosis cutis, *RCM* Reflectance confocal microscopy, *SCN* Subepidermal calcified nodule

Dermoscopy and RCM have been proven more accurate and sensitive than naked-eye examination for detecting skin cancers [17]. Although sensitivity and specificity using these methods cannot be calculated for individual cases, dermoscopy and RCM have suggested some distinctive features of SCN. With an increasing number of cases and the prevalence of noninvasive in vivo imaging, the diagnostic value of dermoscopy and RCM in SCN will be enhanced.

The current treatments for SCN include excision, CO_2_ laser, conservative care, salicylic acid, and intralesional triamcinolone [4]. The majority of cases are managed using excisional methods. There are two reasons for using excision as a first-line treatment option for SCN. Surgical removal, along with histological examination, is the most effective method of simultaneous treatment and confirmation of diagnosis. Additionally, complete surgical excision can help prevent a recurrence. No recurrence was noted in our patient after surgical excision. Sodium thiosulfate (STS) has been reported as another possible treatment for calcinosis cutis [18], particularly in cases with smaller lesions. However, it is unknown whether it can be used in eyelid SCN. If a diagnosis of SCN can be confirmed with dermoscopy and RCM, topical application of STS may become an option. These methods also allow real-time monitoring of its therapeutic efficacy.

In conclusion, clinicians should consider the possibility of SCN for an adolescent patient with a painless yellowish-white papule. The combination of dermoscopy and reflectance confocal microscopy with conventional histopathology helps improve the diagnostic accuracy of SCN.

## Data Availability

The data used and analyzed during the current study are available from the corresponding author upon reasonable request.

## Abbreviations

SCN: Subepidermal calcified nodule
RCM: Reflectance confocal microscopy
